# Timed Up-and-Go as a predictor of fracture risk: a systematic review and meta-analysis in a population of over 1.6 million people

**DOI:** 10.3389/fpubh.2026.1841017

**Published:** 2026-05-11

**Authors:** Jordan Hernandez-Martínez, Izham Cid-Calfucura, Edgar Vásquez-Carrasco, Joaquin Perez-Carcamo, Tomás Herrera-Valenzuela, Pablo Aravena-Sagardia, Mauricio Barramuño-Medina, Pablo Valdés-Badilla

**Affiliations:** 1 Department of Physical Activity Sciences, Universidad de Los Lagos, Osorno, Chile; 2 Department of Education, Faculty of Humanities, Universidad de la Serena, La Serena, Chile; 3 Escuela de Ciencias del Deporte y Actividad Física, Facultad de Salud, Universidad Santo Tomas (UST), Santiago, Chile; 4 Occupational Therapy School, Faculty of Psychology, University de Talca, Talca, Chile; 5 Centro de Investigación en Ciencias Cognitivas, Faculty of Psychology, Universidad de Talca, Talca, Chile; 6 VITALIS Longevity Center, Universidad de Talca, Talca, Chile; 7 Department of Physical Activity, Sports and Health Sciences, Faculty of Medical Sciences, Universidad de Santiago de Chile (USACH), Santiago, Chile; 8 Physical Education Pedagogy, Faculty of Education, Universidad Autónoma de Chile, Temuco, Chile; 9 Kinesiology Program, Faculty of Health Sciences, Universidad Autónoma de Chile, Temuco, Chile; 10 Department of Physical Activity Sciences, Faculty of Education Science, Universidad Católica del Maule, Talca, Chile; 11 Sport Trainer Program, School of Education, Universidad Viña del Mar, Viña del Mar, Chile

**Keywords:** accidental falls, aged, frail older adults, physical functional performance, postural balance

## Abstract

**Objectives:**

This systematic review aimed to synthesize evidence from prospective cohort studies assessing the Timed Up-and-Go (TUG) test as association of fracture risk in older people, and to quantify the association through meta-analysis using hazard ratios (HR).

**Methods:**

Two authors independently conducted systematic searches in PubMed/MEDLINE, Scopus, Web of Science, CINAHL Complete, and the Psychology and Behavioral Sciences Collection. MEDLINE was accessed through PubMed and was therefore not treated as a separate database. The Psychology and Behavioral Sciences Collection was included because it may capture studies addressing behavioral, psychological, and functional factors relevant to fracture risk. Eligible cohort studies were those that examined the association between slower TUG performance, reflected by longer completion time in seconds, and all-cause fractures in older people. HR estimates with 95% confidence intervals (CI) were pooled using random-effects meta-analysis after assessment of between-study heterogeneity.

**Results:**

Two authors independently extracted the data. Six studies with 1,639,397 participants. A low TUG score was associated with hip fractures from all causes (HR = 1.64; 95% CI = 1.20–2.22), as well as with overall fractures (femur, spine lumbar, pelvis, forearm and proximal humerus; HR = 1.38; 95% CI = 1.05–1.80). However, a modest but statistically significant association was observed for vertebral fractures (HR = 1.11; 95% CI = 1.03–1.20).

**Conclusion:**

Poor performance on the TUG test is associated with hip fractures and overall fractures in older people.

**Systematic review registration:**

https://www.crd.york.ac.uk/PROSPERO/view/CRD420251244709, identifier PROSPERO (CRD420251244709).

## 1 Introduction

During ageing, progressive alterations in the vestibular, visual, and proprioceptive systems, together with musculoskeletal degeneration, lead to a decline in postural balance and functional mobility, substantially increasing the fall risk and fractures in older people (1–3). Falls are the second leading cause of death from unintentional injuries worldwide (4), as approximately one in three older people experiences at least one fall each year (5). These events frequently result in fractures, particularly of the lower limb, most notably hip and femoral fractures which are associated with marked morbidity, loss of independence, and excess mortality (6, 7). Beyond their clinical consequences, fall-related fractures impose a substantial economic burden, with direct medical costs exceeding 50 billion USD annually in the United States of America alone (8).

Given this burden, substantial efforts have focused on identifying functional markers capable of predicting fracture risk in older people, with prior studies primarily examining static postural balance measures and reporting inconsistent associations with incident fractures. For instance, Nordström et al. (2), in a cohort of 5,437 self-sufficient older people in Sweden, reported that postural balance assessed using the Nintendo Wii Balance Board predicted hip fracture risk adjusted for sex only in older women, including mediolateral eyes open (HR = 1.01) and eyes closed (HR = 1.10), as well as anteroposterior eyes closed (HR = 1.14) conditions. In contrast, Dovjak et al. (9) in a study of 3,448 geriatric patients admitted to an acute geriatric department in Australia, found that postural balance assessed with the Tinetti balance test was not predictive of overall fracture risk (HR = 0.97).

In contrast, dynamic postural balance and functional mobility are more closely aligned with the real-world circumstances in which falls and fractures occur (10). The Timed Up-and-Go (TUG) test is a simple, low-cost, performance-based measure that integrates essential components of functional mobility, including rising from a chair, walking, turning, and sitting, and is typically performed over standardized distances of 2.44 or 3 meters (11). By capturing the coordinated involvement of sensorimotor (including visual and vestibular inputs) and musculoskeletal systems during a functional task, TUG provides a relevant measure of dynamic balance, with potential prognostic value for fracture risk (12, 13). Poorer TUG performance has been associated with reduced gait independence, increased fall risk, and adverse health outcomes in older people (14), and the TUG test has demonstrated high validity and reliability for 0.91 predicting femoral fractures (15) and, of 0.82 fall risk (16).

Despite the widespread use of the TUG test in both clinical and research settings, and the growing number of longitudinal studies reporting associations between TUG performance and fracture outcomes, this lack of synthesis limits the use of longitudinal evidence in clinical screening and fracture prevention strategies. Although previous studies has examined fracture prediction based on alterations in static postural balance, yielding inconsistent findings (2, 9), no comprehensive analysis has focused on fracture risk using tests that specifically assess dynamic postural balance, such as the TUG (12, 13). Consequently, it remains unclear whether TUG performance consistently predicts fracture risk across different populations and fracture sites, or to what extent observed associations are robust to between-study heterogeneity.

Given the importance of longitudinal evidence for informing clinical practice and government health policy decisions in ageing societies (17), a comprehensive synthesis of the available data is warranted. Therefore, this systematic review aimed to synthesize evidence from prospective cohort studies assessing the TUG test as association of fracture risk in older people, and to quantify the association through meta-analysis using hazard ratios.

## 2 Methods

A systematic review with meta-analysis were conducted following the guidelines of the Cochrane Collaboration. Findings were reported according to the Preferred Reporting Items for Systematic Reviews and Meta-Analysis (PRISMA) (18). The review was registered in PROSPERO (code: CRD420251244709).

### 2.1 Search strategy

Two authors (JH-M and EV-C) conducted a systematic search of the PubMed/MEDLINE, Scopus, Web of Science (core collection), CINHAL Complete, and Psychology and Behavioral Sciences Collection databases up to April 21, 2026. The following terms were used: (“Fractures, Bone”[Mesh] OR “Osteoporotic Fractures”[Mesh] OR “Accidental Falls”[Mesh]) OR (“fracture” OR “fracture risk” OR “fracture incidence” OR “fracture prediction” OR “fracture prognosis”)) AND (“timed up and go” OR “timed up-and-go” OR “timed up and go test” OR “TUG”) AND (“Aged”[Mesh] OR “Frail Elderly”[Mesh] OR “older adults” OR “older people” OR “elderly” OR “aged” OR “geriatric”) AND (“prognostic” OR “predictive” OR “hazard ratio” OR “odds ratio” OR “risk ratio” OR “cox regression” OR “multivariate analysis”). No language restrictions were applied in the study selection process. In addition, the literature search was supplemented through the manual review of reference lists in the selected articles.

### 2.2 Selection criteria

The *a priori* inclusion criteria for this systematic review with meta-analysis were: (i) exposure: postural balance or mobility using the TUG test; (ii) primary outcome: risk of fracture from all causes assessed using hazard ratios (HR-Cox proportional hazards model) associated with low-impact mechanisms (e.g., fragility fractures); (iii) participants: community-dwelling older people ≥ 65 years (19) with preserved functional independence or those with arthritis or osteoporosis; excluding studies in which all participants had more than one chronic disease such as diabetes, heart failure, hypertension, peripheral arterial disease, chronic obstructive pulmonary disease, cancer, and patients with serious illnesses (i.e., studies of patient groups were excluded); and (iv) study design: prospective cohort studies. Two authors (JH-M and PV-B) independently evaluated the results of the electronic search. When the title of an article appeared relevant, the abstract was reviewed to determine its eligibility. When more information was required, the full text of the article was retrieved and evaluated. Any differences in assessments between the two authors were discussed and, if necessary, a third author (TH-V) was involved in the decision-making process. The reasons for excluding identified articles were recorded in all cases. Finally, when two studies used the same sample, the study with the longest follow-up was included.

### 2.3 Data collection process and data elements

Two authors (JH-M and PV-B) independently extracted data such as the name of the first author, year of publication, duration of follow-up, study location, sample size, age of participants at baseline, HR (and associated 95% CI or standard errors), adjusted variables, method of assessment of TUG and fracture risk, outcomes of interest, and number of cases.

### 2.4 Risk of bias in individual studies

Two authors (JH-M and PV-B) independently assessed the risk of bias using the ROBINS-I tool for non-randomized studies, following Cochrane guidance (20). This instrument evaluates seven domains of bias confounding, participant selection, intervention classification, deviations from intended interventions, missing data, outcome measurement, and selective reporting and assigns an overall judgement based on the highest risk identified across domains.

### 2.5 Summary measures

All analyses were performed in R. Hazard ratio (HR) estimates with corresponding 95% confidence intervals (CIs) were extracted from the included studies for each outcome of interest related to fracture risk, and pooled HRs were calculated using random-effects models (DerSimonian and Laird). A likelihood-based random-effects meta-analysis was used to account for between-study variance (21). When HR data were not directly available, corresponding authors were contacted by email, with up to three attempts, to request the missing information. If the full texts were inaccessible or no response was received within 30 days of the last email, the studies were assessed using the available published data and excluded from the meta-analysis when essential data could not be obtained.

### 2.6 Synthesis of results

The percentage of total variation across the studies due to heterogeneity (Cochran’s Q statistic) (22), was used to calculate the *I*^2^ statistics, considering *I*^2^ values of <25, 25–50%, and >50% as small, medium, and large heterogeneity, respectively (23). Sensitivity analysis were conducted to assess the robustness of the summary estimates to determine whether a particular study accounted for the heterogeneity. Thus, each study was deleted from the model once to analyze the influence of each study on the overall results.

### 2.7 Sensitivity analysis

To evaluate the robustness of the findings, sensitivity analysis were conducted for meta-analysis including at least four studies. A leave-one-out approach was applied, whereby pooled effect sizes were recalculated after sequential exclusion of each study using the same DerSimonian–Laird random-effects model. Changes in pooled estimates, statistical significance, and heterogeneity indices (*τ*^2^, *Q*, and *I*^2^) were examined. Additional sensitivity analysis were performed by excluding studies identified as potentially influential or methodologically problematic based on two predefined criteria. First, studies classified as high risk of bias using the ROBINS-I tool were excluded when their removal left at least three studies available for analysis. Second, influential or outlying studies were identified through visual inspection of forest plots and quantitative influence diagnostics (24), including DFBETAS (>|1|), Cook’s distance (>4/n; >0.5 in small samples), and hat values (>2k/n, with k = 1). Studies exceeding one or more thresholds were excluded in supplementary analysis to assess the stability of pooled effects. When all studies contributing to a given outcome were rated as high risk of bias, sensitivity analysis based on study quality were not feasible and robustness was assessed solely through leave-one-out and influence diagnostics.

### 2.8 Risk of bias across studies

Small-study effects bias was assessed using the extended Egger’s test (25), and the presence of publication bias was investigated graphically by funnel plots.

## 3 Results

**Figure 1:**
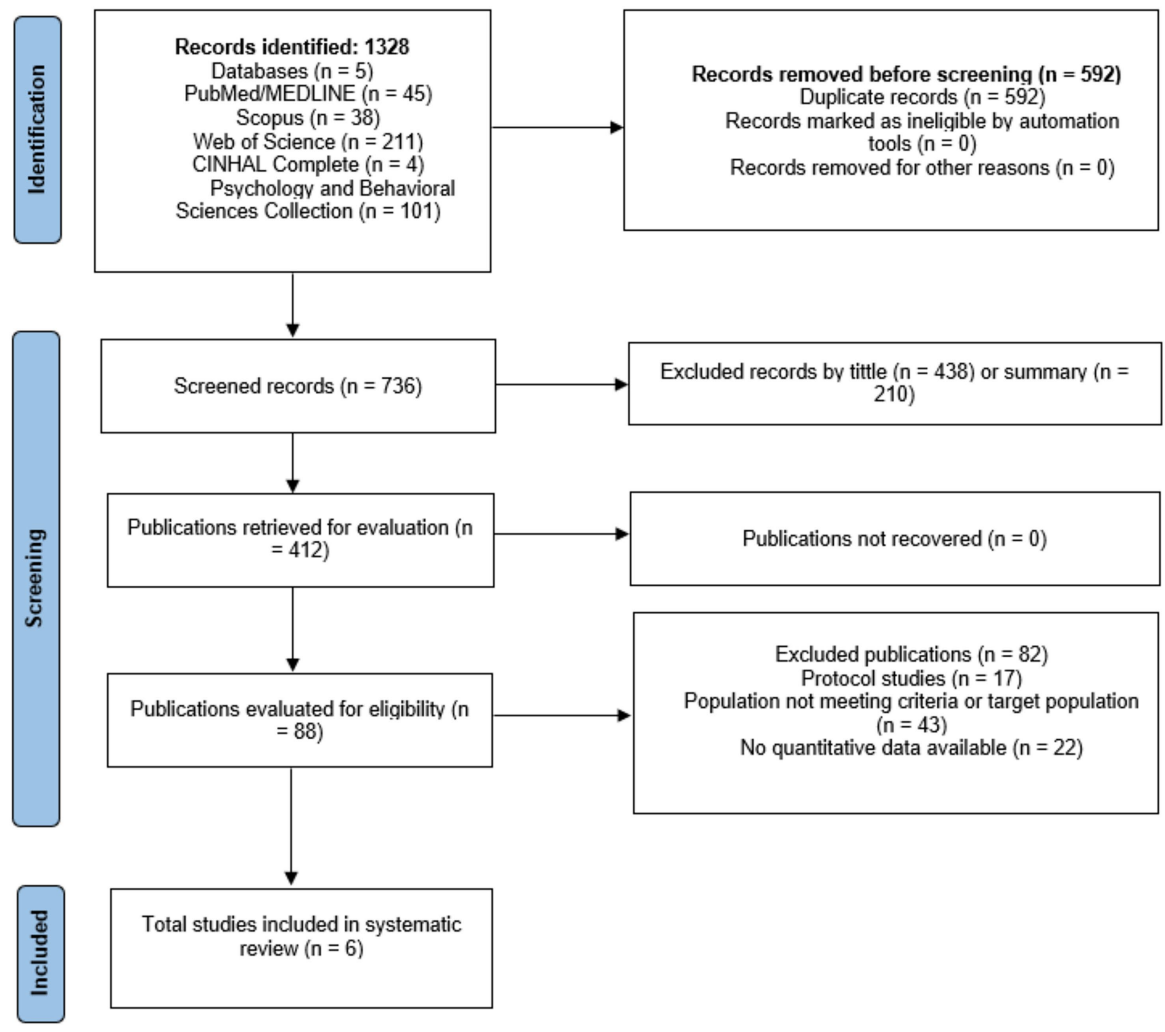
Flowchart of the review process. Based on the PRISMA guidelines (18).

The electronic search strategy retrieved 1,328 studies. After removing duplicate references and based on title (*n* = 438) and abstract (*n* = 210), 738 studies were read in full text. This left 88 articles, of which 17 protocol studies, 43 population not meeting criteria or target population, and 22 not quantitative/incomplete outcome data were eliminated, leaving 6 quantitative studies for the corresponding analysis (26–31). These results are presented in Figure 1.

### 3.1 Study characteristics

Table 1 summarizes the characteristics of the 6 included studies (26–31). The six studies included a total sample of 1,639,397 participants, with sample sizes ranging from 875 to 1,070,320 participants, including both male and female mean aged ≥66 years. Of these studies, three were conducted in Asia (26, 28, 29), and three in Europe (27, 30, 31). Follow-ups ranged from 1 to 7 years, and fracture detection in all studies was performed using X-ray reports in all studies (26–31).

**Table 1:** Characteristics of the studies analyzed in systematic review with meta-analysis.

Authors (year)	Country	Sample size (*n*)	Mean age or range	Male (%)	Female (%)	Predictive fall risk test	Adjustment for covariates	Follow-up
Chun et al. (26)	The Korean National Health Insurance Corporation (KNHIC)	557,648	≥66 years	50	50	TUG	Adjusted for sex, comorbidity, dementia and ADL problems. Adjusted for sex, BMI, comorbidity, dementia, smoking, and alcohol. Adjusted for sex, BMI, comorbidity, dementia, smoking, alcohol and ADL problems.	1 to 4 years
Larsson et al. (30)	SUPER Sahlgrenska University Hospital Prospective Evaluation of the Risk of Bone fractures is a prospective population-based study, carried out in the greater Gothenburg area.	3,028	78.2	0	100	TUG	Adjusted for age, height, and weight. Adjusted for age, height, weight, and clinical risk factors. Adjusted all covariates used in model 2 with the addition of BMD.	1 year
Marques et al. (31)	Age, Gene/Environment Susceptibility (AGES)-Reykjavik Iceland Study with impaired kidney function.	875	76	36.5	63.5	TUG	Adjusted for age and sex. Additionally adjusted for weight, height, percentage of weight change from age 50, physical activity level, cognitive status, history of fracture, diabetes, and glucocorticoid use.	4.3 to 7.3 years
Jeong et al. (29)	National Health Information Database (NHID) of the Korean National Health Insurance Services (NHIS).	1,070,320	≥66	49	51	TUG	Adjusted for sex, body mass index, income, smoking, alcohol consumption, regular physical activity, fall history, and unipedal balance test. Adjusted for the same variables as in model 1 + comorbidities (hypertension, diabetes, chronic kidney disease, cancer) and mental/cognitive function	4.4 years
Hagino et al. (28)	Japan	3,247	74.0	N/A	N/A	TUG	Adjusted for confounding factors (age, T-score, the presence of a prevalent vertebral fracture, the presence of a prevalent non-vertebral fracture, 25(OH)D, family history of hip fracture, diabetes mellitus, and rheumatoid arthritis.	2 years
Gregori et al. (27)	Swedish population and needed to understand Swedish.	3,028	77.5	0	100	TUG	Adjustments for age, body mass index (BMI), FRAX CRFs, femoral neck BMD and all physical function tests as predictors both individually and simultaneously.	

TUG, Timed Up-and-Go; BMI, Body Mass Index; BMD, bone mineral density; FRAX, Fracture Risk Assessment; CRFs, clinical risk factors.

### 3.2 Timed Up-and-Go (TUG) measurement

The methodology applied to evaluate the TUG test in the 6 studies (26–31) was a distance of 3 m measured with a stopwatch, where participants had to get up from a seated position, walk 3 m, turn around, return to the chair, and sit down (11). The cut-off points for establishing reduced mobility in the TUG varied between >10 s (27, 28, 31), ≥12 s (29, 30), and ≥20 s (26).

### 3.3 Risk of bias within studies

**Figure 2:**
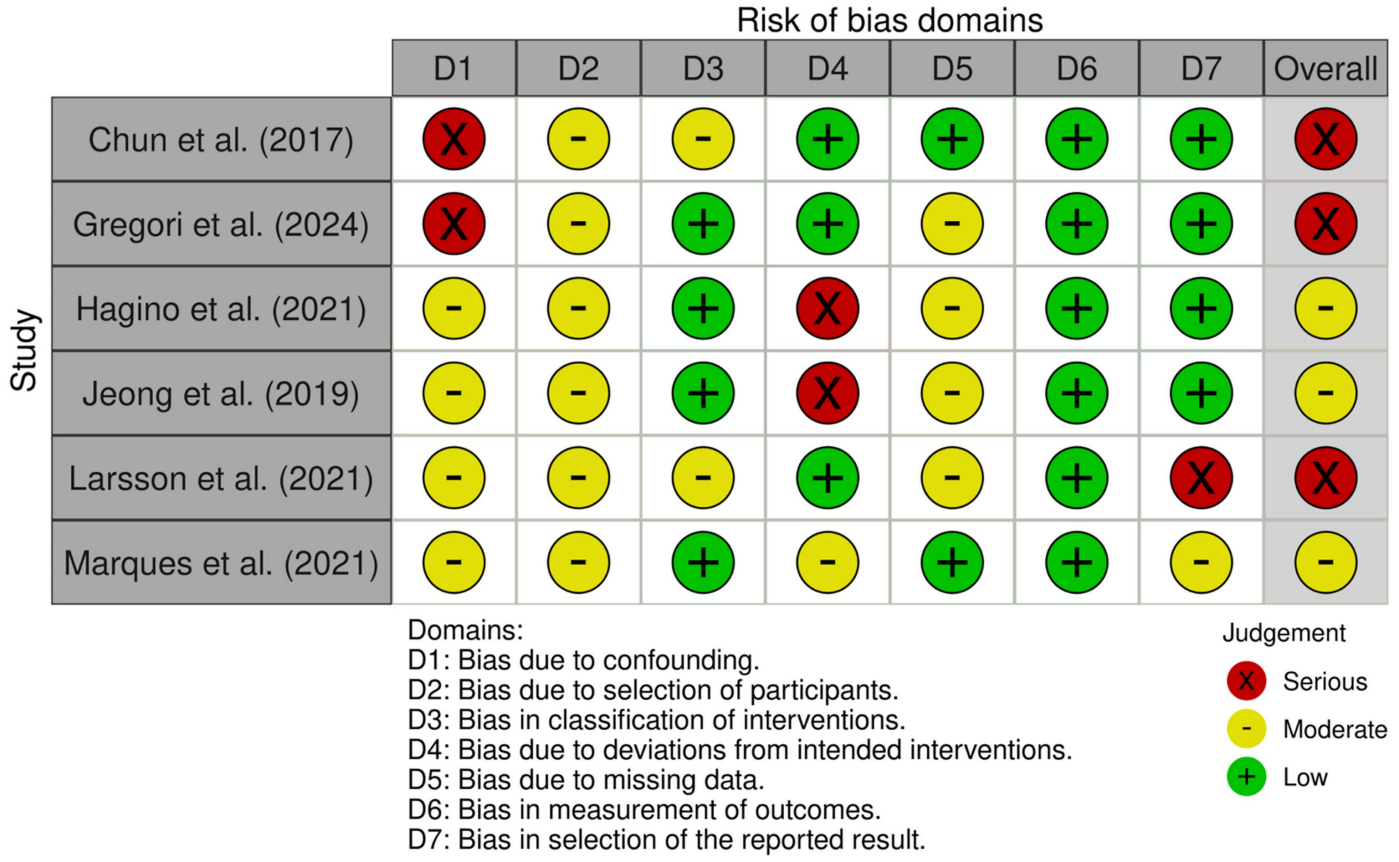
A summary of the risk of bias assessments.

**Figure 3:**
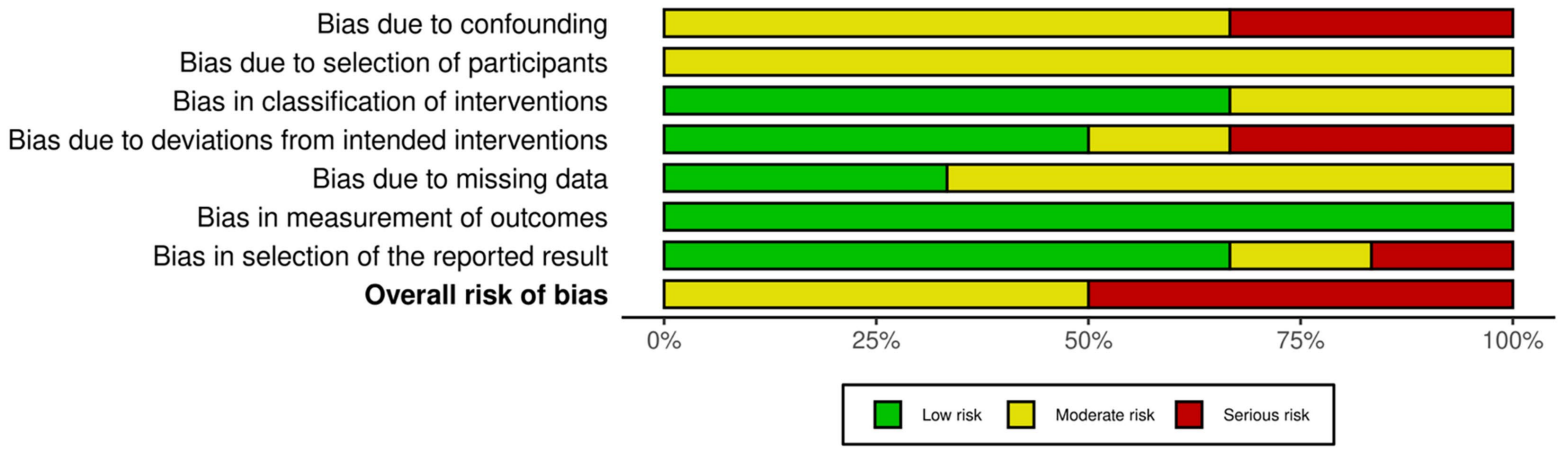
Risk of bias: traffic light graph.

The ROBINS-I risk assessment revealed that the studies presented primarily a serious risk of confounding (D1) and, to a lesser extent, intervention bias (D4), particularly in Chun et al. (26) and Gregori et al. (27). The domains of participant selection (D2), intervention classification (D3), and missing data (D5) predominantly showed a moderate risk, consistent with common methodological limitations in non-randomized designs. In contrast, outcome measurement (D6) and reporting selection (D7) were mostly assessed as having a low risk, indicating standardized and adequate procedures. According to the ROBINS-I criteria, the overall assessment of most studies was classified as having a moderate to serious risk, suggesting evidence susceptible to residual bias and requiring cautious interpretation (Figures 2, 3).

### 3.4 Certainty of evidence

The certainty of evidence was assessed using the GRADE approach based on six observational studies that analyzed the TUG test as a predictor of fractures in older people. The risk of bias was judged as serious, and inconsistency was also considered serious, whereas no relevant concerns were identified regarding indirectness or imprecision, and no other considerations applied. In total, the included studies comprised 1,638,146 participants. Consequently, the overall certainty of evidence was rated as low. The clinical importance of the outcome was considered high, given that the TUG test is a relevant functional measure in the assessment of fracture risk and other adverse musculoskeletal outcomes in older people (Table 2).

**Table 2:** Evaluation of methodological quality using the GRADEpro tool.

Certainty assessment							Number of patients		Effect		Certainty	Importance
Number of studies	Studio design	Risk of bias	Inconsistency	Indirect evidence	Vagueness	Other considerations	Time up and go	[Comparison]	Relative (95% CI)	Absolute (95% CI)		
Timed Up-and-Go as a predictor of fracture in older people												
6	observational studies	serious ^to^	serious	It’s not serious	It’s not serious	none	1,638,146/1638146 (100.0%)		not estimable		Low	IMPORTANT

CI, confidence interval; a. moderate.

### 3.5 Meta-analysis

**Figure 4:**
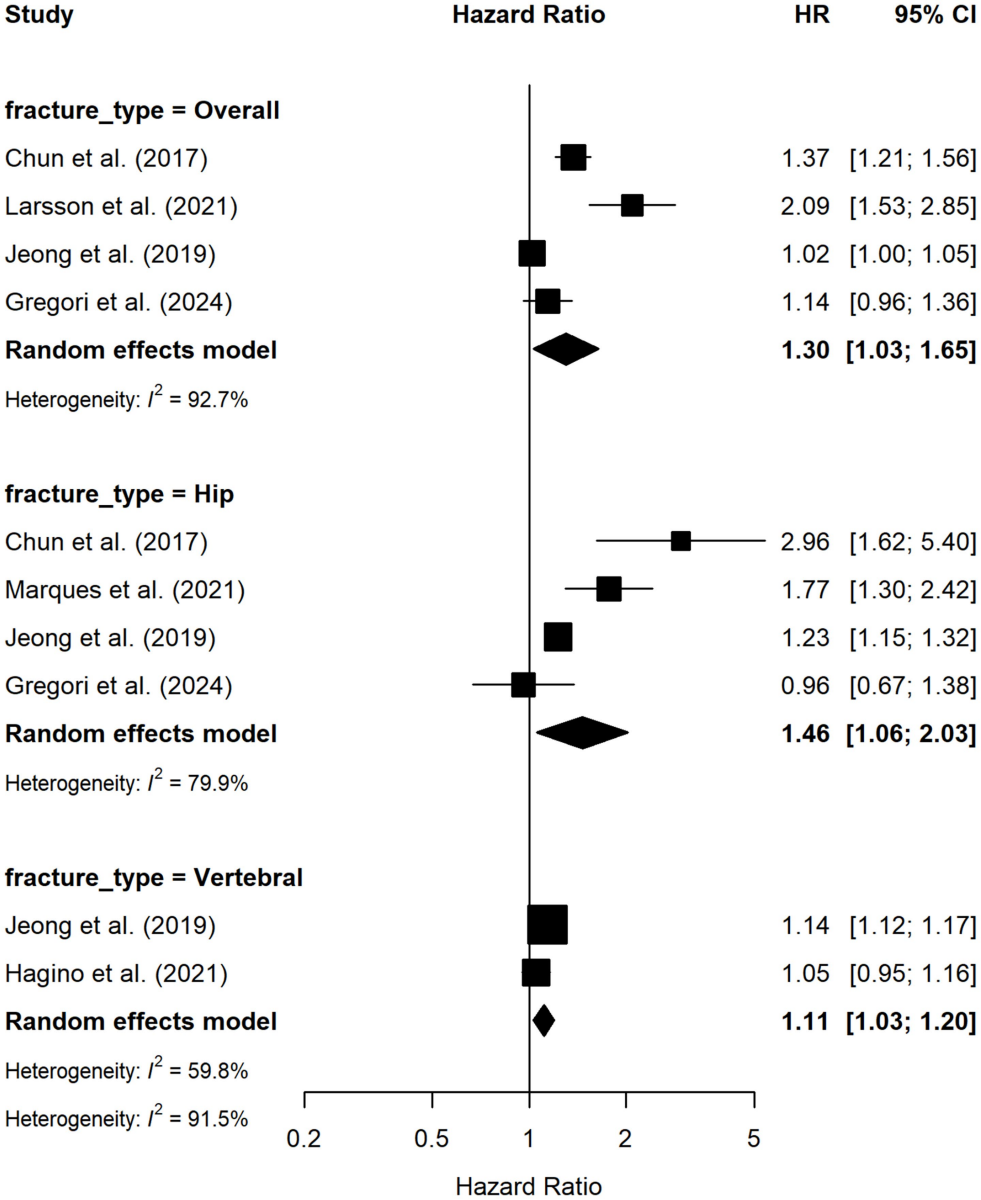
Hazard ratios of low-risk fracture and high-risk fracture measured by TUG. Boxes and horizontal lines represent hazard ratio and 95% CI for each trial. Diamonds represent the 95% CI for pooled estimates of effect. “Fracture_Type = Overall” corresponds to the definition used in the original studies and reflects a composite outcome of multiple fracture sites, including lower limb (femur), lumbar region (lumbar spine and pelvis), and upper limb (forearm and proximal humerus), rather than a pooled estimate across all fracture types. HR, hazard ratio; CI, confidence interval.

Figure 4 shows the HR represent the relative risk of fracture associated with poorer TUG performance, as defined in each individual study low risk (≤1) and high risk (>1) measured using TUG. The TUG test was reported to have a significant association of hip fracture (HR = 1.64, 95% CI = 1.20 to 2.22), with high heterogeneity (*I*^2^ = 79.9%), overall fracture (HR = 1.38, 95% CI = 1.05 to 1.80), with high heterogeneity (*I*^2^ = 92%), and vertebral fracture (HR = 1.11; 95% CI = 1.03 to 1.20) with moderate heterogeneity (*I*^2^ = 58%). The results of the heterogeneity are presented graphically in Supplementary Figures S1–S3.

### 3.6 Publication bias and sensitivity analysis

Visual inspection of the funnel plots for TUG outcomes (in Supplementary material) did not reveal clear evidence of marked asymmetry for overall or hip fracture. However, the small number of included studies limits the ability to reliably assess publication bias or small-study effects. For vertebral fracture, interpretation of funnel plot symmetry was further constrained by the inclusion of only two studies. Therefore, while no obvious indication of publication bias was observed, these findings should be interpreted with caution.

Regarding overall fracture risk, a significant association with TUG performance was observed (HR = 1.30; 95% CI = 1.03–1.65; *p* < 0.001), with substantial heterogeneity (*I*^2^ = 92.7%). Sensitivity analysis showed that exclusion of Larsson et al. (30) attenuated the association (logHR = 0.15; HR ≈ 1.16, 95% CI = −0.05 to 0.35) and rendered it non-significant (*p* = 0.143), while heterogeneity remained high (*I*^2^ = 90.6%). In contrast, exclusion of Jeong et al. (29) strengthened the pooled effect (logHR = 0.36; HR ≈ 1.43, 95% CI = 0.10 to 0.62; *p* = 0.0066) and reduced heterogeneity (*I*^2^ = 82.2%), suggesting a disproportionate contribution of this study to between-study variability. Removal of other studies did not materially affect the pooled estimates or heterogeneity. For hip fracture risk, TUG performance was also significantly associated with fracture risk (HR = 1.46; 95% CI = 1.06–2.03; *p* = 0.002; *I*^2^ = 79.9%). Exclusion of Larsson et al. (30) (logHR = 0.25; HR ≈ 1.28, 95% CI = −0.02 to 0.51; *p* = 0.066) or Marques et al. (31) (logHR = 0.33; HR ≈ 1.39, 95% CI = −0.09 to 0.75; *p* = 0.126) resulted in loss of statistical significance, whereas exclusion of Jeong et al. (29) yielded a larger but still non-significant effect (logHR = 0.50; HR ≈ 1.65, 95% CI = −0.07 to 1.07; *p* = 0.086). In contrast, exclusion of Gregori et al. (27) strengthened the association and preserved significance (logHR = 0.54; HR ≈ 1.71, 95% CI = 0.11 to 0.97; *p* = 0.014), with heterogeneity remaining substantial (*I*^2^ = 84.4%). For vertebral fracture risk, only two studies were available, and no significant association with TUG performance was observed (HR = 1.11, 95% CI = 1.03–1.20; *p* = 0.115; *I*^2^ = 58.0%). Sensitivity analysis was not feasible, as exclusion of a single study would have precluded estimation of between-study variance.

## 4 Discussion

This systematic review aimed to synthesize evidence from prospective cohort studies assessing the TUG test as a predictor of fracture risk in older people, and to quantify the association through meta-analysis using hazard ratios. Our meta-analysis identified a significant association between overall fracture risk and TUG performance (HR = 1.38; 95% CI = 1.05–1.80). Similarly, hip fracture risk was significantly associated with TUG performance (HR = 1.64; 95% CI = 1.20–2.22). In contrast, vertebral fracture risk showed a statistically significant but modest association with TUG performance (HR = 1.11; 95% CI = 1.03–1.20; *p* = 0.115; *I*^2^ = 58.0%). However, the substantial heterogeneity observed across most analysis reflects important methodological and clinical differences between studies, including variability in TUG cut-off points, follow-up duration, covariate adjustment strategies, and population characteristics such as sex distribution, baseline functional status, and fracture risk profiles.

Our findings are consistent with those reported by Nordström et al. (2), who showed that postural balance impairments such as increased body sway velocity and greater variability in sway velocity were associated with a higher fracture risk in both male and female aged 70 years. To our knowledge, this is the first study to use the TUG test as a predictor of fracture risk in older people and to quantify this association through a meta-analysis using HR. The results of our study suggest that poorer TUG performance is associated with an increased risk of overall fractures and hip fractures. However, the substantial heterogeneity observed across most analysis reflects important methodological and clinical differences between studies, including variability in TUG cut-off points, follow-up duration, covariate adjustment strategies, and population characteristics such as sex distribution, baseline functional status, and fracture risk profiles.

On the other hand, sensitivity analysis showed that although the direction of the association between TUG performance and fracture risk was generally consistent, the statistical significance of the pooled estimates particularly for overall and hip fractures was sensitive to the exclusion of individual influential studies (27, 29–31). These findings indicate that, while the direction of the association between TUG performance and fracture risk is consistent across studies, the magnitude of the effect and its statistical significance vary depending on the study included, thereby warranting a cautious interpretation of our findings.

Impairments in postural balance constitute a significant fall risk factor in older people (32), and are characterized by the interaction of multiple potentially modifiable components, including gait and balance disorders, orthostatic hypotension, sensory deficits, cognitive and psychological factors (e.g., executive dysfunction, attentional deficits, fear of falling), the use of certain medications, and environmental hazards (2, 33, 34). In this context, fractures occur predominantly as a consequence of falls that take place during the performance of dynamic and everyday functional tasks, such as rising from a chair or walking (2). These actions represent critical functional demands in activities of daily living in older people and correspond directly to the components assessed by the TUG (14). In particular, the transition from sitting to standing requires an anticipatory displacement of the center of pressure, followed by its acceleration in both the anteroposterior and mediolateral directions, as well as adequate postural control to achieve stabilization in an upright position process that may be compromised with ageing (14, 35). Beyond the initial postural transition, the TUG requires appropriate gait initiation, as well as acceleration and deceleration during walking and preparation for performing consecutive turns (14, 35). These specific components of the TUG may be particularly relevant to the biomechanics of falls leading to hip fractures. In particular, impaired turning, reduced control of gait speed, and difficulties during deceleration and transitional movements may compromise mediolateral stability and increase the likelihood of sideways falls, which are commonly associated with hip fractures in older people (36, 37). In this context, poorer TUG performance may reflect not only a general reduction in functional mobility, but also specific deficits in dynamic balance and movement coordination that are directly linked to fracture-prone fall mechanisms. Both the initial turning sequence and the final turn prior to sitting have been described as particularly challenging tasks, even for healthy older people over 70 years, and to an even greater extent for frail older individuals with mild postural balance impairments (38). In this context, shorter TUG completion times (<10 s) have been reported to be strongly associated with gait independence in older people (39), whereas a cut-off value of 13.5 s has been proposed as a threshold for identifying individuals at increased fall risk (14).

From a preventive perspective, identifying older people at increased fall risk through tests such as the TUG is particularly relevant in the context of interventions aimed at improving balance, mobility, and functional capacity. Available evidence suggests that multicomponent training programs combining strength exercises, balance, and functional tasks are effective in reducing fall risk in this population (40–42). Within this framework, innovative interventions such as exergames (43) and other task-based training modalities with combat sports (44) have shown promising results by simultaneously stimulating motor, sensory, and cognitive components involved in postural balance. Therefore, the use of the TUG as a functional assessment tool may help identify older people with greater impairments in dynamic postural balance and mobility who could potentially benefit from multiprofessional interventions targeting these domains, with possible implications for reducing the fall risk and fractures.

### 4.1 Limitations and strengths

Our meta-analysis has several limitations that should be considered when interpreting the findings: (i) the number of included studies was limited, particularly for vertebral fracture outcomes, precluding sensitivity analysis and restricting the robustness of inferences for this fracture type; (ii) substantial heterogeneity was observed across most analysis, likely reflecting methodological and clinical differences between studies, including variability in TUG cut-off points, follow-up duration, covariate adjustment, and population characteristics such as sex distribution, baseline functional status, and fracture risk profiles; (iii) sensitivity analysis showed that the statistical significance of pooled estimates for overall and hip fractures was sensitive to the exclusion of individual influential studies, indicating that the available quantitative evidence remains context-dependent; and (iv) this review mainly represents relatively healthy older people, which may limit the generalizability of the findings to typical geriatric populations characterized by multimorbidity. Future prospective studies are needed to determine whether the predictive value of the TUG test remains consistent in more vulnerable geriatric populations, particularly among older people with multimorbidity, frailty, and reduced functional reserve, who are underrepresented in the current evidence base. Although all included studies were prospective cohorts, residual risk of bias cannot be entirely excluded, particularly regarding functional performance assessment and residual confounding. While the TUG protocol is simple and widely standardized, minor differences in its implementation may also have contributed to the observed heterogeneity.

In contrast, the strengths of our meta-analysis include: (i) being the first meta-analysis to systematically evaluate the TUG test as a predictor of fracture risk in older people using HR derived exclusively from prospective cohort studies; (ii) a comprehensive search strategy across major multidisciplinary databases, including PubMed/MEDLINE, Scopus, Web of Science, CINAHL Complete, and the Psychology and Behavioral Sciences Collection. The search syntax was tailored to each database in order to optimize the sensitivity and specificity of the strategy; (iii) adherence to rigorous methodological standards, including PRISMA guidelines, ROBINS-I for risk of bias assessment, and GRADE for evaluating the certainty of evidence; and (iv) the application of leave-one-out sensitivity and influence analysis to assess the robustness of the results and to identify studies with a disproportionate impact on pooled estimates, thereby enhancing the transparency and interpretability of the findings.

### 4.2 Practical applications

From a clinical and public health perspective, the TUG test can provide relevant functional information on mobility and dynamic postural balance in older people, with implications for identifying individuals at higher risk of hip fracture. Nevertheless, considering the observed heterogeneity and the moderate certainty of evidence, TUG performance should not be interpreted in isolation. Its primary practical value lies in complementing multifactorial fracture risk assessment strategies and guiding referral to preventive interventions aimed at improving mobility, balance, and functional capacity, with possible indirect implications for reducing the fall risk and fractures in older people.

From an epidemiological perspective in public health for older people, the TUG test represents a viable and low-cost functional measure that can be incorporated into population assessments of older people in different community centers, helping to provide valuable information when integrated into multifactorial fracture risk models with low performance on this test, facilitating risk stratification and allowing the identification of subgroups with impaired mobility and dynamic balance who could benefit from specific preventive strategies. Therefore, TUG is a complementary screening tool that can help inform resource allocation and guide referrals to community-based exercise and rehabilitation interventions aimed at improving functional capacity and potentially reducing fall-related outcomes among older people.

## 5 Conclusion

Poorer performance on the TUG test is associated with an increased fracture risk in older people, particularly hip fractures, albeit with considerable heterogeneity and a moderate certainty of evidence. Although the direction of the association was consistent, the statistical significance of the pooled estimates was sensitive to influential studies, underscoring the need for cautious interpretation of the findings. In this context, the TUG emerges as a potentially useful functional tool to contribute to multifactorial fracture risk assessment in older people.

## Data Availability

The original contributions presented in the study are included in the article/Supplementary material, further inquiries can be directed to the corresponding author.
