# Supplementary material for: Timed Up-and-Go as a predictor of fracture risk: a systematic review and meta-analysis in a population of over 1.6 million people
Source: Front Public Health. 2026 May 11;14:1841017. doi: 10.3389/fpubh.2026.1841017 (PMC13199231; doi:10.3389/fpubh.2026.1841017)
Supplement: Supplementary file 1 [file Data_Sheet_1.PDF]

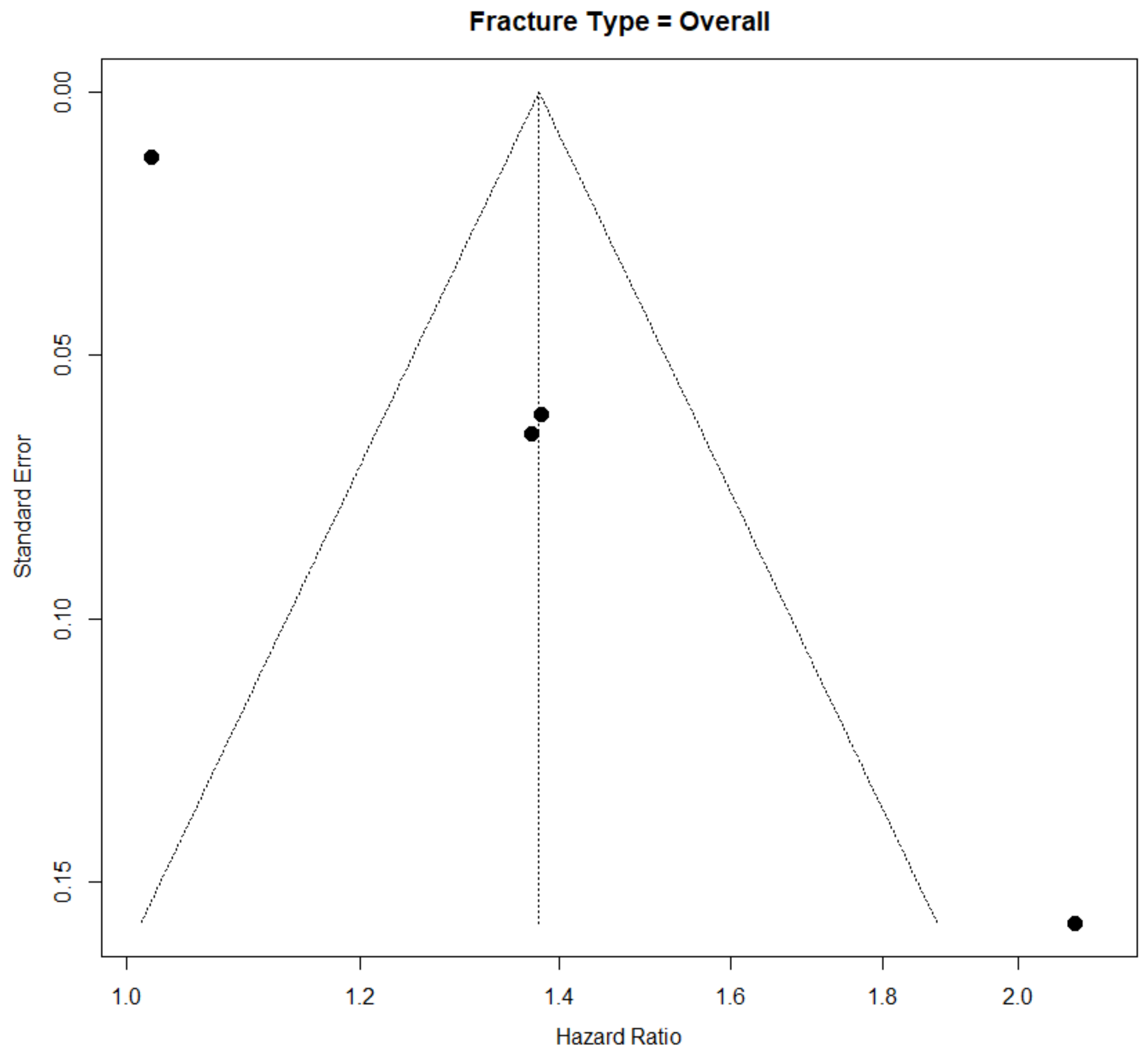

Figure S1. Funnel plots TUG publication bias for overall fractures.

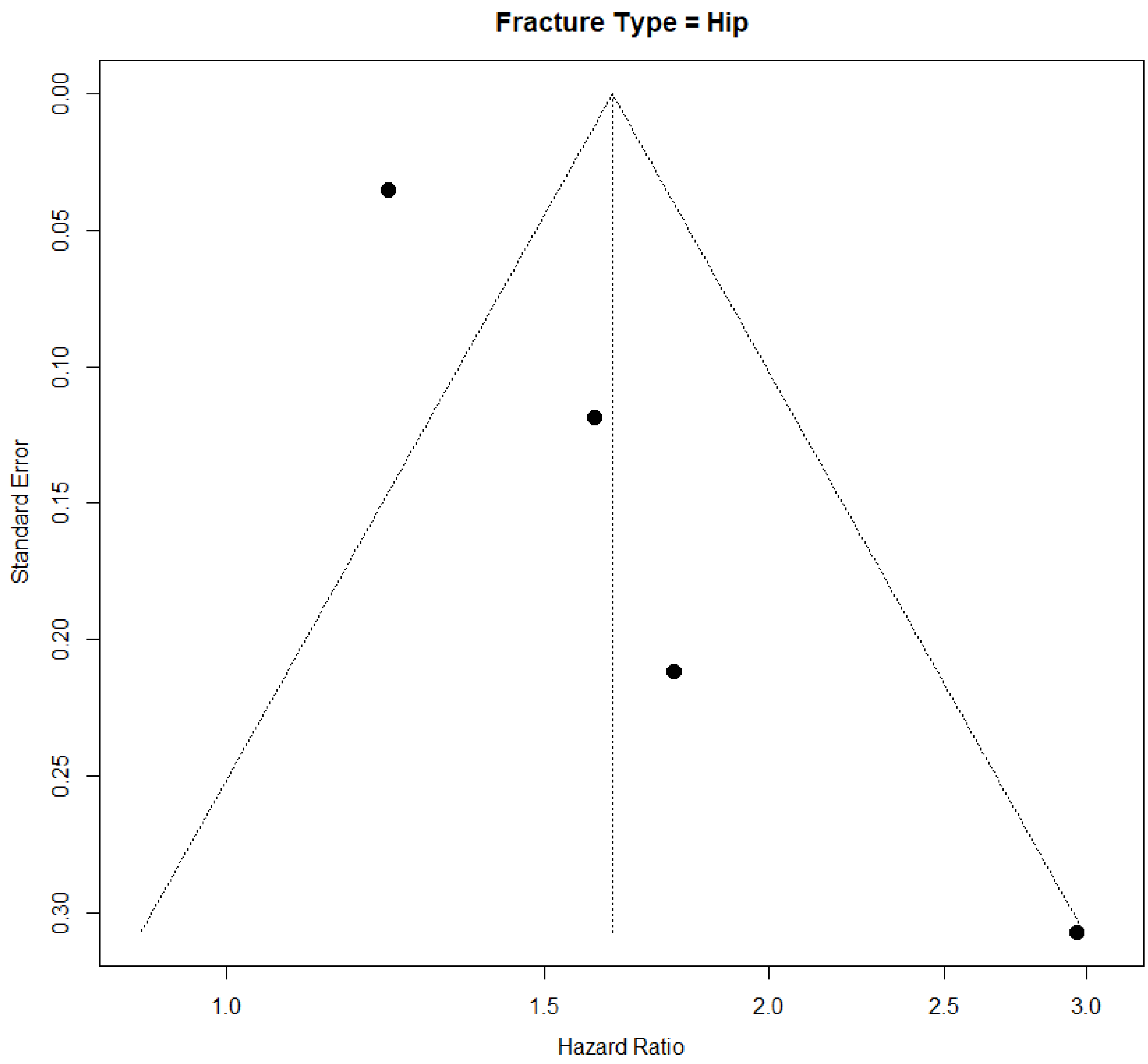

Figure S2. Funnel plots TUG publication bias for hip fractures.

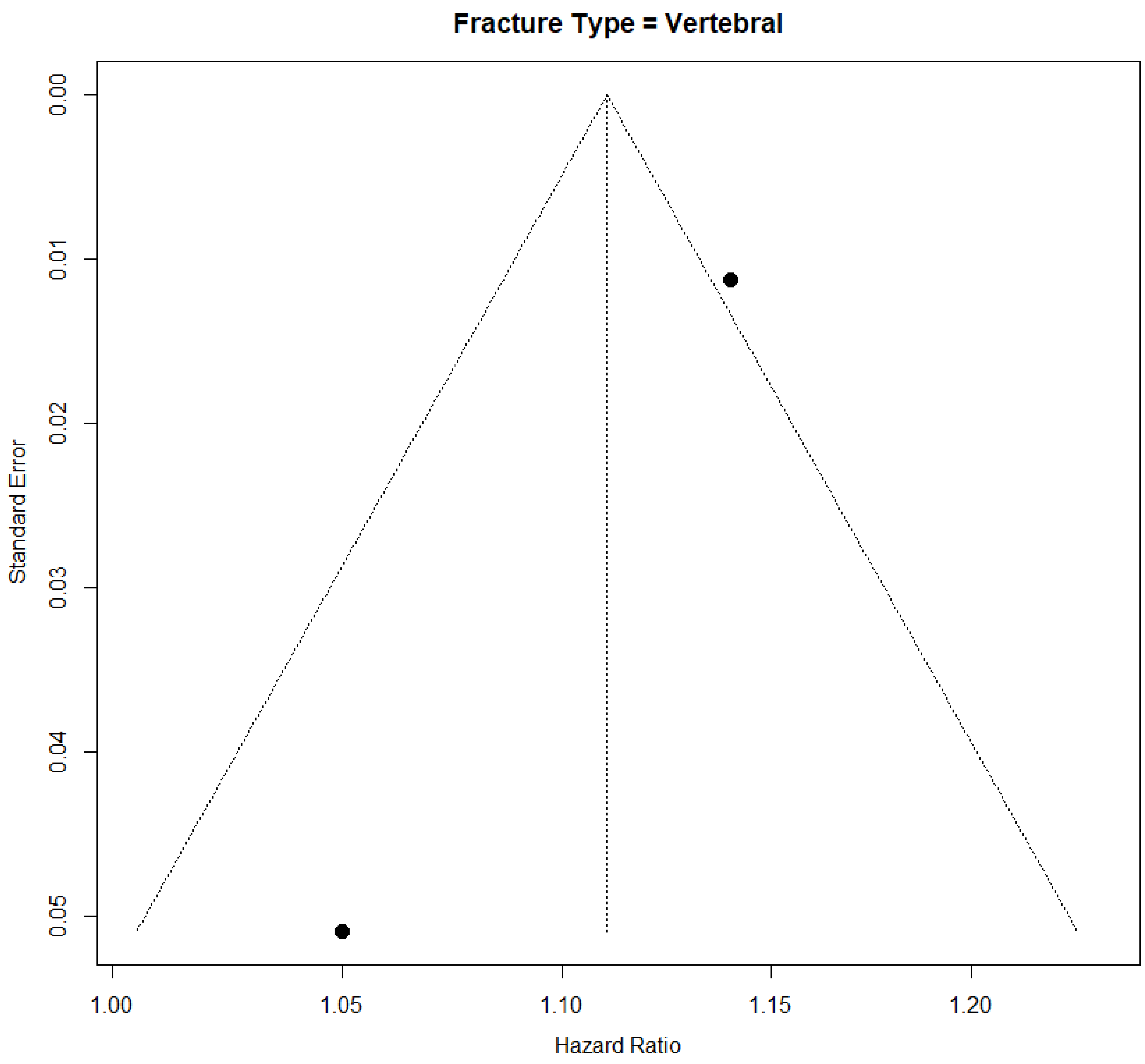

Figure S3. Funnel plots TUG publication bias for vertebral fractures.
